# Microbial control measures for soft ice cream in franchise brands: A comparative analysis of microbial analysis and manufacturing practices

**DOI:** 10.1002/fsn3.1446

**Published:** 2020-02-21

**Authors:** Jong Myong Park, Jong Mun Kim, Ji Won Hong, Young‐Hyun You

**Affiliations:** ^1^ Department of Infectious Disease Diagnosis Incheon Institute of Public Health and Environment Incheon Republic of Korea; ^2^ Food Safety Center LOTTE Group Global R&D Center Jakarta Republic of Indonesia; ^3^ Department of Taxonomy and Systematics National Marine Biodiversity Institute of Korea Seocheon Republic of Korea; ^4^ Microorganism Resources Division National Institute of Biological Resources Incheon Republic of Korea

**Keywords:** food franchise, food safety management, good manufacturing practice, ice cream, microbial safety, third‐party hygiene audit

## Abstract

The aim of this study was to establish a complementary direction of the franchise food hygiene guideline to control microbial risks. We evaluated current measures of preventing microbial spoilage and ensuring microbiological safety of food in the food franchise industry. Manufacturing practices were assessed using microbiological analyses, third‐party food safety audits, and existing hygiene guidelines. Microbial load indicators of food, manufacturing processes, work environment, and workers were also analyzed to track microbial proliferation. We audited manufacturing practices and processes and analyzed the cleaning and sanitation clauses. We found high contamination of heterotrophic bacteria and detected coliforms in some products. There was no direct contamination by food handlers, and the sterility of raw materials was satisfactory. The main issues were structural complexities of equipment, which hindered cleaning and disinfection, and disinfection practices of franchise brands. Store‐level microbial control may be compromised due to the industrial nature of small stores operating collectively. We thus issued complementary guidelines. Improved collective microbiological safety may be ensured through implementation of the revised cleaning and sterilization regulations.

## Graphical abstract

Microbial load indicators of food, manufacturing processes, work environment, and workers were analyzed to track microbial proliferation. Manufacturing practices and processes were audited. Cleaning and sanitation guidelines were analyzed. Complementary guidelines were issued for highly developing food franchise industry.

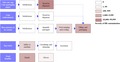

## 1 INTRODUCTION

Rapid changes in socioeconomic and demographic factors affecting the Korean food industry environment are changing its industrial structure (Park et al., 2019). The food industry must always be prepared to meet consumer demands (Aliouche & Schlentrich, 2011; Andrew, Madhav, Michael, & Oliver, 2016, Song, 2013). In addition to the manufacturing industry, which is linked to the development of the existing Korean food industry, the service industry—a tertiary industry—has also rapidly developed in association with the food industry (Park et al., 2019). The franchise industry is a representative example of the service‐based food industry (Andrew et al., 2016; Park et al., 2019; Van der Wagen & Goonetilleke, 2015a, 2015b). Franchises are geographically dispersed in Korea, and some have also been established abroad (Park et al., 2019; Park, Kim, & Nam, 2017). Nevertheless, an optimized operating system maintains a high degree of homogeneity in the quality of foods and services provided by individual stores (Aliouche & Schlentrich, 2011; Gary & Rasheed, 2003; Gills & Castrogiovanni, 2012; Van der Wagen & Goonetilleke, 2015a, 2015b). These franchises grow by securing brand identities based on various features (Khanm, 1991). 

A new industrial group that is developing rapidly must secure a food safety management strategy for sustainable growth (Blay‐Palmer, 2016). Any food accident, such as poisoning, which occurs while a franchise is growing or trying to advance into foreign markets (Saigoneer, 2018; Vietnamnet, 2018), will not only damage the franchise's image but also affect the entry of other franchises (Park et al., 2019). Any such newly evolving franchise industry will inevitably lag behind traditional, well‐developed food industries in developing safety management measures. Established effective systems such as HACCP, which are applied to food processing and manufacturing (Luning & Marcelis, 2006; Trienekens & Zuurbier, 2007; Varzakas, 2011), have characteristics that are not well suited for application in franchises, due to certain differences in business operation and work environments (Park et al., 2019). However, homogeneous operating characteristics may help food franchises at least maintain their processes at a uniform level (Aliouche & Schlentrich, 2011; Gary & Rasheed, 2003; Gills & Castrogiovanni, 2012; Lora & Douglas, 2005; Van der Wagen & Goonetilleke, 2015a, 2015b). Well‐prepared management guidelines that ensure uniform processes may act as cornerstones that raise the food safety management status of entire franchisee stores.

Although many microbiological surveys of the final products of food service businesses have been conducted, studies aimed at creating microbial management practices that target the manufacturing process are rare (Park et al., 2019). Therefore, we conducted a microbiological assessment of soft ice‐cream manufacturing processes, which are basic and display typical process characteristics of leading franchises. Furthermore, as soft ice creams are products that remain unsterilized following the final steps of processing, their microbial loads are difficult to control (Leisnter & Gould, 2002).

The overall aim of this study was to establish a complementary direction of the franchise food hygiene guideline to control microbial risks based on the comparative analysis of our data from manufacturing process analysis, microbiological analysis during the manufacturing process, analysis of existing food safety guideline per each franchise brand, and third‐party food safety audits. In particular, the aim of the manufacturing process analysis is to systematically conduct microbiological analysis and third‐party audit at each stage of the process. The analysis of existing guidelines is intended to identify weaknesses that need to be supplemented based on microbial and audit results. The aim of the third‐party audits was to accurately determine where and why microorganisms are introduced and proliferate throughout the food process stage. By comparing and analyzing the results, we will be able to identify critical causes and fundamental countermeasures against microbial contamination. We hope to lay the foundation for food safety management in the highly growing, service‐based food industry.

## 2 MATERIALS AND METHODS

### 2.1 Sampling principles

Sampling involved the following criteria: (a) All samples were obtained without previous notification; (b) stores with limited raw materials that were temporarily without sales or that had no customers at the time of sampling were excluded; and (c) all samples were delivered within 3 hr of being sealed and maintained at 4°C for immediate testing. All samples were analyzed in triplicate. Samples were collected three times a day at the same time over a 3‐month period during the summer season when stores were most at risk for microbial contamination or manipulation. Another reason for sampling during the summer was that soft ice‐cream houses produce and sell more types of chilled products in the summer than in the winter. Therefore, it was considered more appropriate to collect samples during the summer instead of throughout the year.

### 2.2 Sampling materials

Microbial growth in food facilities may be promoted by various factors, including inadequate utensils, tools, machinery, food handler control, and time–temperature control (Gould, Rosenblum, Nicholas, Phan, & Jones, 2013). Thus, the current microbial analysis was directed at factors that affect microbial loads of final products. Raw materials used were ice‐cream premixes, cone confectionaries, and water used for cleansing (Table 1, sample number 1–3), and yield products were ice cream that was in the process of being made inside the machine (Tables 1, 2, 3). Final products encompassed products that were ready for customer consumption (Table 1, sample number 5), equipment and tools (inside surface of ice‐cream makers, ice‐cream emissions parts, outlet surface of makers, storage utensils for cones, quantitative cups used to make ice cream, take‐out cups, take‐out cup dispensers) (Table 1, sample number 6–11), and food handlers (hands and disposable gloves) (Table 1, sample number 12, 13). In summary, each of the 14 samples were collected three times from 10 brand franchisee stores, for a total count of 420 samples.

**Table 1:** Number of franchisees showing microbial contamination

Category		Sample number	Target item	Heterotrophic bacteria	Coliform bacteria	*Escherichia coli* †	*Staphylococcus aureus* †
Food products and materials	Raw materials	1	Ice mix	–	–	–	–
		2	Cone confectionary	–	–	–	–
		3	Water for cleansing	5	–	–	–
	Yield materials	4	Ice cream under overrun	3	–	–	–
	Final products	5	Soft ice cream	6	1 (10%)	–	–
Processing environment	Machinery, utensils, packaging	6	Inside surface of ice‐cream maker	1	–	–	–
		7	Outlet surface of ice‐cream maker	3	–	–	–
		8	Storage utensils for cone confectionary	4	–	–	–
		9	Utensils used for mixing ice	1	–	–	–
		10	Ice cream take‐out cup	2	–	–	–
		11	Storage utensils for take‐out cups; packaging paper	4	–	–	–
	Food handler	12	Hands	6	1 (10%)	–	–
		13	Disposable gloves	3	–	–	–

Note

**Figure 1:**
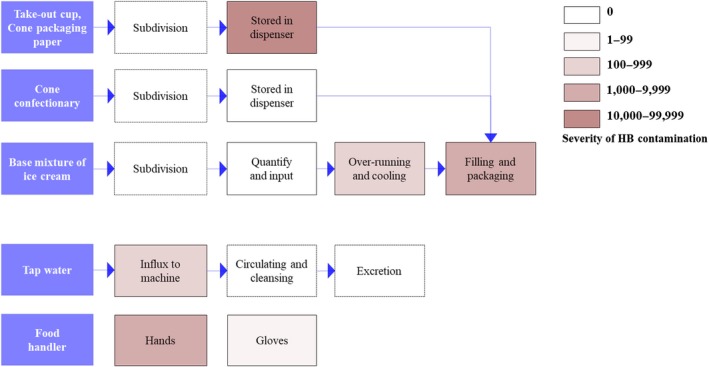
Results of process analysis and heterotrophic bacteria (HB) density. Increasing color intensity indicates the increasing severity of HB contamination of materials or yield products. The results are also shown in Table 2. HB contamination increased as processing progressed

HB density data from this table was analyzed and visualized in Table 2 and Figure 1 (process chart) to trace microbial influx or microbial manipulation as the manufacturing process progressed.

^†^ *S. aureus* or *E. coli* were not detected in any workplaces, food materials, or food handlers.

**Table 2:** Number of franchisees showing HB microbial contamination

Category		Sample number	Target item	ND	1–99 CFU/ml	100–999 CFU/ml	1,000–9,999 CFU/ml	10,000–99,999 CFU/ml
Food products and materials	Raw materials	1	Ice mix	10	–	–	–	–
		2	Cone confectionary	10	–	–	–	–
		3	Water for cleansing	5	3 (30%)	2 (20%)	–	–
	Yield materials	4	Ice cream under overrun	7	2 (20%)	1 (10%)	–	–
	Final products	5	Soft ice cream	4	2 (20%)	2 (20%)	2 (20%)	–
Processing environment	Machinery, utensils, packaging	6	Inside surface of ice‐cream maker	9	1 (10%)	–	–	–
		7	Outlet surface of ice‐cream maker	7	2 (20%)	1 (10%)	–	–
		8	Storage utensils for cone confectionary	6	1 (10%)	–	2 (20%)	1 (10%)
		9	Utensils used for mixing ice	6	2 (20%)	2 (20%)	–	–
		10	Ice cream take‐out cup	7	1 (10%)	1 (10%)	–	1 (10%)
		11	Storage utensils for take‐out cups; packaging paper	5	4 (40%)	1 (10%)	–	–
	Food handler	12	Hands	4	2 (20%)	3 (30%)	1 (10%)	–
		13	Disposable gloves	7	2 (20%)	1 (10%)	–	–

Note

HB contamination increased as the processing steps progressed. Increasing intensity of the color indicates increasing severity of HB contamination of materials, yield, and final products.

Abbreviation: HB, heterotrophic bacteria.

**Table 3:** Microbiological analysis of food, associated environment, packaging materials, and food handlers

Category		Sample		Microorganism	Establishment									
					1	2	3	4	5	6	7	8	9	10
Food products and materials	Raw materials	Ice mix	1	HB	–	–	–	–	–	–	–	–	–	–
				Coliform	–	–	–	–	–	–	–	–	–	–
		Cone confectionary	2	HB	–	–	–	–	–	–	–	–	–	–
				Coliform	–	–	–	–	–	–	–	–	–	–
		Water for cleansing	3	HB	–	–	–	0.30 ± 0.02	2.45 ± 1.02	2.18 ± 1.00	1.89 ± 0.46	1.03 ± 0.06	–	–
				Coliform	–	–	–	–	–	–	–	–	–	–
	Yield materials	Ice cream under overrun	4	HB	1.01 ± 0.24	2.00 ± 0.18	–	–	3.73 ± 1.70	–	–	–	–	–
				Coliform	–	–	–	–	–	–	–	–	–	–
	Final products	Soft ice cream	5	HB	1.00 ± 0.00	1.48 ± 0.06	2.00 ± 1.00	2.08 ± 1.22	3.96 ± 1.90	3.78 ± 2.48	3.73 ± 1.62	3.18 ± 1.70	0.70 ± 0.24	‐
				Coliform	–	–	–	–	1.01 ± 0.51	–	–	–	–	–
Processing environment	Machinery, utensils, packaging materials	Inside surface of ice‐cream maker	6	HB	1.01 ± 0.24	–	–	–	–	–	–	–	–	–
				Coliform	–	–	–	–	–	–	–	–	–	–
		Outlet surface of ice‐cream maker	7	HB	1.07 ± 0.24	1.19 ± 0.24	–	2.04 ± 1.22	–	–	–	–	–	–
				Coliform	–	–	–	–	–	–	–	–	–	–
		Storage utensils for cone confectionary	8	HB	1.07 ± 0.24	–	–	3.04 ± 1.70	3.05 ± 1.83	–	4.02 ± 1.22	–	–	–
				Coliform	–	–	–	–	–	–	–	–	–	–
		Utensils for mixing ice	9	HB	1.29 ± 0.24	1.71 ± 0.36	–	2.73 ± 1.40	2.78 ± 0.88	–	–	–	–	–
				Coliform	–	–	–	–	–	–	–	–	–	–
		Ice cream take‐out cup	10	HB	–	1.59 ± 1.44	2.93 ± 1.70	–	4.04 ± 1.84	–	–	–	–	–
				Coliform	–	–	–	–	–	–	–	–	–	–
		Storage utensils for take‐out cups; packaging paper	11	HB	–	3.54 ± 1.85	3.38 ± 1.70	1.66 ± 0.58	4.18 ± 2.70	–	–	–	–	–
				Coliform	–	–	–	–	–	–	–	–	–	–
	Food handler	Hands	12	HB	2.06 ± 0.79	1.05 ± 0.24	2.09 ± 0.54	1.05 ± 0.24	–	–	–	–	2.13 ± 1.16	3.05 ± 1.69
				Coliform	–	–	–	–	–	–	–	–	–	–
		Disposable gloves	13	HB	1.04 ± 0.00	–	–	–	–	–	–	–	0.94 ± 0.24	2.00 ± 0.64
				Coliform	–	–	–	–	–	–	–	–	–	–

Note

Results are expressed as the mean ± standard deviation. *Staphylococcus aureus* and *Escherichia coli* were not detected on any targets and are thus not included in this table.

Abbreviation: HB, heterotrophic bacteria.

### 2.3 Microbial analysis

Indicator microorganisms including heterotrophic bacteria (HB), *Staphylococcus aureus*, coliforms, and *Escherichia coli* were analyzed (Rossi, Beilke, & Barreto, 2017). Quantitative analyses of the four indicator microorganisms were performed for all targets. Each sample was collected from the surface of a listed work environment (Tables 1 and 2; *n* = 10) or the surface of a worker's hand and disposable glove (*n* = 20). To collect samples, sterile swabs moistened in tubes filled with sterile phosphate buffer were rubbed on the surface. A 10 × 10 cm mold (100 cm^2^) was used to delimit the area of collection. The swab, rubbed on the 10 × 10 cm surface in three different directions, was placed in a test tube containing 10 ml sterile 0.1% phosphate‐buffered saline (Rossi et al., 2017). HB were counted using a standard plating method (Rossi et al., 2017). Plate counting agar (5 g tryptone, 2.5 g yeast extract, 1 g dextrose, 15 g agar; PCA; Merck) was sterilized at 121°C for 15 min. One milliliter of each homogenized sample was diluted 10^5^–10^6^ fold with sterile 0.85% saline and incubated at 36 ± 1°C for 48 hr. The number of coliform bacteria was determined using Violet Red Bile Agar (Merck) incubated at 36 ± 1°C for 24 hr. The presence of coliforms was confirmed by transferring characteristic colonies (pink halo) into tubes containing *E. coli* (E.C) broth (Merck) and incubating at 45 ± 0.2°C for 48 hr. The most probable number of coliforms was determined using the multiple‐tube fermentation technique with brilliant green bile broth and E.C. broth. To analyze *E. coli*, 1 ml of test solution was inoculated into three tubes containing E.C. medium and cultured at 44.5 ± 0.2°C for 24 hr for qualitative analysis. If gas was generated, the result was considered positive; otherwise, it was considered negative. Samples deemed positive were incubated on eosin methylene blue agar (Merck)‐containing medium at 36°C for 24 hr, following which typical colonies were implanted in lactose broth and nutrient agar. The lactose medium was incubated for 48 hr at 36°C and then cultured at 36°C for 24 hr. If the lactose medium generated gas, a colony from the corresponding agar medium was selected and classified as gram‐negative and nonspore forming. Quantitative tests were not performed because suspected colonies of *E. coli* were not detected during the qualitative tests (Rossi et al., 2017). Swabs from food handlers were subjected to qualitative analyses for *S. aureus*, while quantitative tests were not performed. *S. aureus* was detected using 0.1 ml of sample via the spreading plate technique on Baird‐Parker Egg Yolk‐tellurite agar (Merck). The plate was incubated at 36°C for 48 hr, after which characteristic colonies (black with halos) were subjected to gram staining and biochemical tests for confirmation (Rossi et al., 2017). In general, *E. coli* are analyzed as part of coliform testing; however, because the Korean food safety Act or Korean food standard code defines criteria for *E.* *coli* or coliforms obtained from equipment and various food categories, the analyses were conducted using those parameters. Although the criteria for detecting *E. coli* and coliforms are specified for each food product, food utensil, or device, the purpose of this study was to determine the processes or environments from which these microbes originated or moved to. Thus, all products and environments were tested for coliform bacteria.

### 2.4 Third‐party food safety audit

#### 2.4.1 Professional auditing

Audits were conducted by third parties to ensure objectivity and expertise (Ollinger, Moore, & Chandran, 2004; Viator, Muth, Brophy, & Noyes, 2017). Auditors who conducted third‐party audits had at least 6 years of experience (third part auditing, education, checklist development on food safety), have relevant degrees, and are published in professional international journals on food safety management.

#### 2.4.2 Checklist development

Generally, the checklist used in this project was developed by checking for the most basic compliance with the country's laws (Luning & Marcelis, 2006; Trienekens & Zuurbier, 2007; Varzakas, 2011). Moreover, in‐depth criteria, recommended widely as global guidelines, were also used to develop the checklist (British Retail Consortium, 2011; Chaifetz & Chapman, 2015; Chilled Food Association, 2006). Checklist clauses related to microbial control issues applied by this study are listed in Table 4. The checklist content presented in this study largely consisted of 7 categories and 22 sub‐categories. All checked items consisted of factors affecting the microbial status of manufacturing processes (Table 4). These included (a) evaluation of sanitation and cleaning practices to assess whether the franchise brand operates their own sanitary guidelines, including process operation, cleaning, and sterilization, and whether all workers were familiar with these guidelines and performed cleaning and sterilization accordingly. If the cleaning standard, cycle or method was not specified or habitually performed by the operator, actual cleaning and sterilization would be made difficult, thereby affecting microbial contamination; (b) evaluation of utensils and tools to examine whether all food utensils and tools in use were easy to wash or sterilize. Improperly cleaned or disinfected equipment may affect microbial contamination; (c) evaluation of chemicals used as disinfectants and cleaners to assess whether the workers maintained the effective cleaning and disinfecting powers of the chemicals used for cleaning and sterilizing. Appropriate storage and dilution of cleaning and sterilizing agents affects their effectiveness, as less effective agents may fail to control microbial contamination; (d) evaluation of food material management via an assessment of hygienic treatment of raw materials, ingredients, and food packaging materials. Treatment of these materials affects the microbial load of the final product if not hygienic; (e) assessment of processing management via evaluation of all items ranging from warehousing to inspection, processing, packaging, and customer service, because if sanitary conditions of the process are poor, it will affect the microbial load of the final product; (f) assessment of pest control practices via evaluation of insect management throughout the entire workplace, including the manufacturing process. Insect presence directly or indirectly causes microbial contamination or transmission of food‐poisoning bacteria; and (g) inspection of food handlers, as improper management of worker hand washing, sterilization, or working gloves affects microbial loads.

**Table 4:** Manufacturing practices associated with cleaning, sanitization, and microbial control in food workplaces or manufacturing processes

1	Evaluation of sanitation and cleaning practice	Audit result
1.1	Does an individual business perform the specified cleaning/sanitization process for the facility, utensil, tools, or devices?	Some devices are not subject to cleaning and sterilization in their guidelines
1.2	Are all employees familiar with cleaning and sanitization practice?	All workers are aware of the washing and sterilizing targets listed in their brand's manual
1.3	Do all employees conduct cleaning and sanitization processes based on their manual?	All workers performed cleaning and sterilization according to the method described in their brand's manual
1.4	Are all employees familiar with the manufacturing process?	All workers are clearly aware of the work process and process conditions
1.5	Does the manufacturing of products follow prescribed manufacturing processes?	Ensured that all products are manufactured as described in the manual
2	Evaluation of food facility, utensil, tools	Audit result
2.1	Are the device tools made of materials that are easy to clean and sterilize?	Equipment tools that are structurally too complex to clean and sterilize have been identified for each brand's store
2.2	Is the structure of the device or tool easy to clean and sterilize?	All tools were easy to clean and sterilize
2.3	Is the cleaning and sterilizing practically performed?	Substantial cleaning was difficult to perform on devices with structures that were not easily cleaned and sterilized
3	Evaluation of chemicals (disinfectant and cleaner)	Audit result
3.1	Are the cleaning chemicals and disinfectants usable for the food and authorized?	The chemicals used for cleaning and sterilizing we certified and the cleaning and sterilizing agent in use was valid
3.2	Was the cleaning chemical and disinfectant used within the expiration date?	All cleaning and sterilizing agents were used within the expiration date
3.3	Is the dilution method for cleaning disinfectant well managed?	The effective concentration of all cleaning and sterilizing agents was properly managed
4	Food materials management	Audit result
4.1	Is inspection of received food materials conducted?	Inbound inspection of the food materials was carried out properly
4.2	Does the organization comply with the expiration date of the materials, prevent direct sunlight, and maintain storage temperature?	The expiration date and storage temperature of food materials are properly managed
4.3	Is the material storage area well ventilated and kept clean?	The sanitary conditions of the food and materials storage area were good
5	Processing management	Audit result
5.1	Is the temperature of the process maintained well?	The temperature control of the process was effective
5.2	Is the possibility of cross‐contamination with other processes well controlled?	There is no possibility of cross‐contamination with other processes
5.3	Are the packing materials and packing materials storage areas designated as clean areas?	The setting of the packing material storage area has been well implemented
5.4	Are the packing materials and packing materials storage areas clean?	The sanitary conditions of the packing materials and packing materials storage areas were insufficient in some establishments
6	Pest control	Audit result
6.1	Is insect pest control in the facility managed through the application of chemicals by a professional company?	Insect control programs were carried out and commissioned by a professional company
6.2	Does the workplace have insect pests and has it improved upon discovery?	No harmful insects or rodents have been identified
7	Food handler	Audit result
7.1	Do food handlers wash and sterilize their hands properly at the start of work and when returning to work?	All workers wore disposable gloves, and the possibility of cross‐contamination by hand is low
7.2	Does the director perform health and infection control of food handlers?	Directors managed medical check‐up results of all food handlers according to legal standards. The health status of the worker is monitored daily, and the worker is excluded in cases where visible symptoms are detected

Note

The audit report results of each practice can be comparatively analyzed with the microbial analysis results described in Tables 1 and 2, and Figure 1.

#### 2.4.3 Field auditing

Audits were conducted according to established checklists. In case of further unusual observations related to microbial control, record taking, and on‐site interviews were separately performed by the auditor (Park et al., 2019). If needed, interview content obtained from individual franchisee stores were re‐verified via interviews with their franchise headquarters. With regard to processes involving chilling, all auditors used infrared thermometers (830‐T1; TESTO). The internal or surface temperatures of the product or machinery were checked (Table 4, checklist clause 5.1). An effective chlorine concentration meter (Q‐CL501C; ES TECH,) was used to check the concentration/dilution of a disinfectant. Temperature and effective chlorinated concentrations were measured three or more times using a standard protocol to verify that they did not deviate from the reference criteria. Furthermore, food safety management guidelines of each franchisee's own brands were analyzed by auditors for the purpose of comparing the results of microbial analysis and third‐party food safety audit (Chaifetz & Chapman, 2015).

#### 2.4.4 Process analysis

Manufacturing process analysis was conducted to detect where microbes that contaminate food processes originated from and investigate microbe multiplication during the total food preparation process. Process analyses were performed at the audit stage of this study (Figure 1).

## 3 RESULTS AND DISCUSSION

### 3.1 Process analysis

Analyses of the processing procedures and the working environment were intended to accurately determine whether microorganisms that affect the microbial load of the final product originated and proliferated in raw materials, processes, environment, or workers (Figure 1) (Park et al., 2019). Manufacturing processes and conditions were well defined in the guidelines of each brand, and these were followed by food handlers of franchised stores (Table 4, checklist clause 1.1–5). Data obtained from third‐party audits indicated that the raw materials, such as liquid or powdered ice mix form, used to manufacture soft ice cream was supplied by the franchises’ headquarters as designated food material. Most of these raw materials were manufactured on a consignment basis by the franchise system. As opposed to conventional commercial ice cream, which passes through a hardening, filling, and packing process (VanWees, Rankin, & Hartel, 2019; Warren & Hartel, 2018), soft ice cream is supplied to the end consumer at −3 to −5°C without undergoing such a process. To impart a smooth texture to ice‐cream mixes (VanWees et al., 2019; Warren & Hartel, 2018), franchise ice‐cream mixes are treated with more nonsolid and presolid fat‐free milk ingredients than are commercialized hard ice cream. If the raw material is a powder, water from a separate quantitative cup is added as needed (blending process). The stirring fan in the cooler is set to a slow rotation, and the temperature is maintained at −6 to −8°C for overruns, which is ensured by audit step (Table 4, checklist clause 5.1). Then, ice cream is squeezed into a cone confectionary or take‐out cup for sale to consumers. Liquid material is divided into separate quantitative cups as necessary and placed in a cooler with a stirring fan that rotates, without any addition of water or milk. When the consumer places an order, the workers habitually wear disposable gloves (Table 4, checklist clauses 1.2, 5.2), retrieve the ice cream from the machine, and sell it. The audit step confirmed that all food handlers associated with ice cream related procedures wore disposable gloves (Table 4, checklist clauses 1.2, 5.2, 7.1, 7.2). Cones or take‐out cups are retrieved from supply packaging and kept in a separate storage container (Table 4, checklist clause 5.3). Cleansing of ice‐cream making machines and inner fans using tap water is set up for automatic cleaning regulated by electronic programs once every 3–4 days. This cleansing process is followed by all establishments (Table 4, checklist clauses 2.2, 2.3). At the time of this study, the inner tube from the ice‐cream cooler to the outlet is cleaned using water. However, sterilization of cooling and mixing parts of machines must be performed manually by food handlers by spraying alcohol‐based disinfectants (Table 4, checklist clauses 1.2, 1.3). In some businesses, ice‐cream discharge outlets exposed directly to the outer environment are sterilized several times a day with alcohol‐based disinfectants by workers wearing sterile disposable gloves (Table 4, checklist clauses 1.2, 1.3). However, many other businesses did not do so, wiping with tissues only or with a dishcloth. Cleaning and sterilizing were also not routinely scheduled (Table 4, checklist clause 2.3).

### 3.2 Manufacturing practice and microbial load of materials and products

*Staphylococcus aureus* and *E. coli* were not detected in any raw material (Table 1), processed products, or final products. Although coliforms were detected in the final product in 1 of 10 franchised stores (Table 1), it was likely not due to cross‐contamination caused by food handlers, as the coliform detected in the product was from a different store than the one in which coliform was detected on a food handler's hands (Table 3). Detection of coliforms indicates fecal contamination (Dufour, 1977).

**Figure 2:**
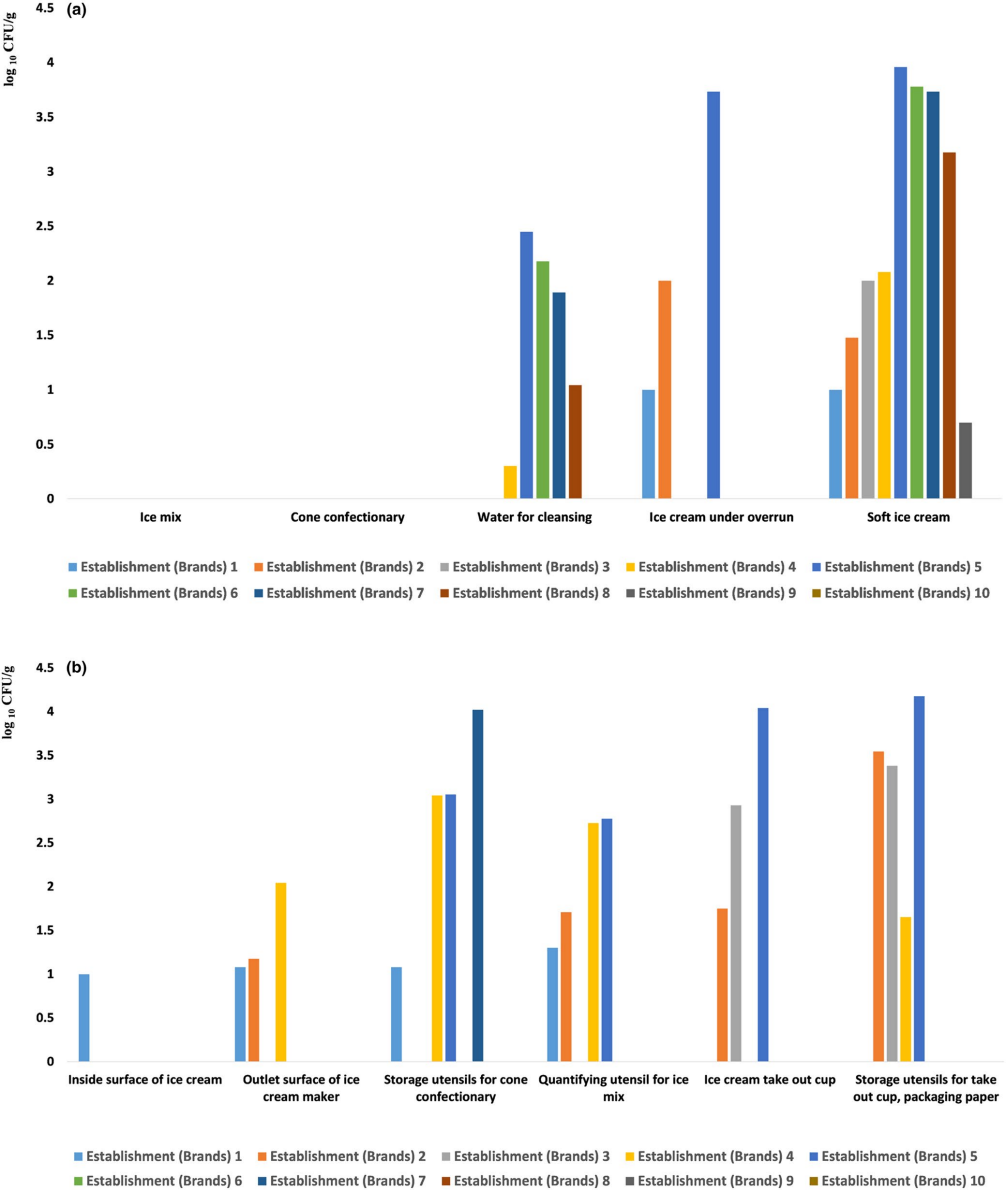
HB load variation per food manufacturing procedures or associated environment. (a) Variation in HB load in food obtained by each processing procedure. As the manufacturing process progressed, the level of HB density rose sharply. Moreover, contamination of the tap water used as cleansing water for the machinery was found to be serious. As the degree of HB contamination inside surface of the ice‐cream maker is low, it was assumed that cleaning and disinfection were properly carried out by food workers. However, contamination of the final product made by this machine means that it is difficult to clean and sterilize parts of the machinery that require disassembly for cleaning. (b) Microbial load of food machinery, utensils, and tools used for food production. Unexpectedly, the hygiene of the inside surface of the ice‐cream machine was ideal, but cleaning and disinfection of the auxiliary and packaging materials or packing materials storage area were poor

As no microorganisms were detected in raw materials such as ice‐cream premixes or cone confectionaries (Tables 1 and 2, Figure 2a), the raw materials are believed to be in a very sterile condition. However, HB were found to gradually increase while ice‐cream mixes were being added to the ice cream maker for manufacturing purposes (Tables 2 and 3, Figure 1, Figure 2a). HB were detected in yield (intermediated) products of ice cream in three franchise stores and their concentrations included 10 colony‐forming units (CFU)/ml, 110, and 5,400 CFU/ml in the franchisees (Table 3). In contrast, final products contained 9,120, 1,500, 5,410, 6,002, 120, 101, 30, 10, and 5 CFU/ml HB (Table 3). The values obtained did not exceed the national standards stipulated by the Korean Food Standard Code (MFDS, 2018a, 2018b). However, given the severity of variation between stores for intermediate products, it appears that management of cleaning and sterilization procedures was differed between franchisee stores (Table 3). In particular, franchisee stores with 5,410 CFU/ml in their yield products exhibited increased HB concentration (Table 2, Figure 1) in the final products (9,120 CFU/ml; Table 2, Figure 1). Coliform counts in one store (10 CFU/ml) exceeded the national microbial safety standards for food (Table 1) (MFDS, 2018a, 2018b). This poses a question of whether final products that do not exceed the HB standard for bacteria are safe. One may contend that ice cream may be considered safe only on the days sampling was conducted. However, if the fundamental management, cleaning, and disinfection criteria are not perfect, problems can occur on other days for the reasons described below. Although analysis of the water used for cleaning ice‐cream makers showed no coliforms or food‐poisoning bacteria, many exceeded the level recommended by Korea's drinking water safety standards for general HB, which is 100 CFU (280, 150, 78, 11, and 2 CFU/ml; Tables 2 and 3, Figure 1) (MOE, 2018). Washing water is delivered via tap water pipes to the actual production part of ice‐cream makers via the machines' own supply piping. Therefore, no matter how thoroughly sterilized the inside of the ice‐cream makers are, the final microbial status of the ice cream may be affected by contaminated cleaning water. This concept may be similar to that associated with hazard analyses and critical control point (HACCP) systems used in traditional manufacturing and processing businesses (Leisnter & Gould, 2002) and food franchises. The water inside the city's water pipes is directly mechanically connected to the ice‐cream maker and thus could not be investigated. However, because typical urban tap water in Korea contains residual chlorine (Liu et al., 2018), it is considered microbiologically safe (Liu et al., 2018). Nevertheless, biofilm formation by microorganisms on the surface of water supply tubes of food appliances makes it difficult to ensure microbiological safety owing to the resistance of biofilms to disinfectants (Ryu & Beuchat, 2005). Moreover, the pipes that supply tap water to ice‐cream makers are difficult to clean and sterilize (Table 4, checklist clause 2.2), and the formation and scale of accumulation of biofilms were visually confirmed during the auditing stage (Table 4, checklist clause 2.3) and microbial analysis (Table 3). Thus, it was concluded that periodic replacement of pipes supplying the ice‐cream machines is necessary. However, for all franchisee brands, neither the franchises' own guidelines nor the management manual of the machine itself described such a practice.

As no *E. coli* were found in any ice‐cream mix raw materials, coolers, internal ice‐cream products, outlets, cones, or packaging materials at any individual store (Table 1), the eventual detection of coliforms in final ice‐cream products can only be explained by contamination in the pipes connecting the cooler to the part of the ice‐cream maker that discharges product. It was also observed that cleaning and disinfecting the discharge tubes, which are not easily dismantled by an operator (Table 4, checklist clause 2.2), is problematic. Overall, it appeared that difficulties in dismantling equipment may lead to them becoming a source of microbial contamination. Disinfectants used to sterilize equipment or workers’ hands were audited at the time of sampling. Auditors confirmed that because food grade commercialized ethanol based chemicals were used (Table 4, checklist clauses 3.1, 3.3), sterilizing agents and handlers’ practices did not pose a threat. There was no deterioration in disinfectant quality due to not complying with expiration dates or not using proper storage conditions (Table 4, checklist clauses 3.2, 3.3).

### 3.3 Manufacturing practices and microbial load of the facility and utensils

Food‐poisoning bacteria, such as *S. aureus* and *E. coli*, were not detected in any of the devices (Table 1). An unusually high density of HB was detected in ice‐cream cups and cup storage boxes (Table 2, Figure 1, Figure 2b). Devices that store cones in some franchisees exhibited an increase in HB concentration (Tables 1 and 3). This may indicate the possibility of contaminated food materials stored in them. No separate cleaning and sterilization cycles were established for storage containers of cones or some ice cream topping confectionaries by most establishments (90%) (Table 4, checklist clause 1.1), regardless of the presence or absence of microbial detection (Tables 1 and 2). Auditors noted that utensils for storing cones were easy to clean and sterilize, as these were not structurally too complex for handlers to disassemble and clean (Table 4, checklist clause 2.2). However, auditors remarked that ice‐cream cup dispensers are structurally too complex for disassembly by food handlers (Table 4, checklist clause 2.2), which could result in serious contamination due to accumulation of foreign substances (Table 2, checklist clause 2.3). Using flashlights, auditors identified a number of foreign deposits inside dispensers, and microbiological analysis of these deposits indicated major HB contamination in several establishments (Table 4, Figure 1). The audit also showed that insect management complied with legal standards (Table 4, checklist clause 6.1) and that insect pests were not found in any workplaces (Table 4, checklist clause 6.2). However, contamination by debris and dust that had accumulated inside the cup dispenser was a serious impediment (Table 4, checklist clause 2.3). Auditors noted that standards had not been established for disassembly and sterilization of cup dispensers across all franchisee stores (Table 4, checklist clause 1.1). Auditors further reported that food handlers lacked knowledge on how to disassemble the dispensers and that some dispensers were impossible to disassemble. Thus, such situations may lead to the production of microbial pollutants and accumulation of foreign substances.

HB were not detected on the inside surface of ice‐cream makers (Table 2, Figure 1). However, sanitary evaluation of discharge cocks that are directly exposed to the outer environment showed HB concentrations of 1,200 CFU/ml in one store (Tables 1 and 2). Auditors further identified that cleaning and disinfecting criteria for discharge parts differed from brand to brand, and that in many stores cleaning and sterilization were dependent only on the memory and habits of some food handlers, instead of relying on the special cycles stipulated by the guidelines. Even the execution of these cycles was dependent on the worker's knowledge of hygiene, rather than being designated as targets for cleaning and sterilizing via the franchisees’ own guidelines. Therefore, it is evident that structural problems of ice‐cream making devices and incomplete disinfection guidelines may lead to increased microbial loads of products.

### 3.4 Manufacturing practices and microbial load of food handlers

Neither *S. aureus* nor *E. coli*, which indicate toxicity (Dagnew, Tiruneh, Moges, & Tekeste, 2012; Park, Cho, Kim, & Ghim, 2018), was detected in any franchisee stores. Coliforms were detected on the hands and gloves of an operator of one store (10 CFU/ml; Table 2, Figure 1). However, it may be natural for workers to carry some HB on their hands. Therefore, this study focused on the fact that even though the HB levels found on the surface of workers hands was denser, the number of bacteria found on the surface of gloves worn by workers was much less dense (Figure 1). Before starting the audit, the auditor assessed the work status of the food handler without prior notice, and all who wore disposable gloves confirmed that they were working (Table 3, checklist clauses 1.2, 7.1). However, considering that the concentration of HB on operators who had worn single‐use gloves for a long period of time (confirmed by on‐site interviews as part of the audit process) was higher than that of other workers, it was concluded that the possibility of cross‐contamination may increase due to the intermittent removal and wearing of disposable gloves for too long.

Furthermore, considering the nature of the franchise, cross‐contamination of the soft ice‐cream manufacturing process by other food processing procedures was a concern. Several parts of the soft ice‐cream manufacturing process rely on machines optimized for fast circulation. As soft ice‐cream manufacturing processes are physically different from other processes, microorganisms may be transferred from other food processes via operators only (Table 4, checklist clause 5.2). Nevertheless, microbial inflow from other processes may not be much of a concern as operator hand hygiene was relatively well managed (Table 4, checklist clause 1.2).

### 3.5 Recommendation for manufacturing practices: cleaning and disinfection

Generally, contamination of food manufacturing processes by microbes may fall into three categories: inflow through raw materials, contaminated utensils/environment, and workers. However, inflow through raw materials was not observed in this study. Therefore, microbial inflow appears to be mostly result from the contamination of equipment, given that workers exhibited positive hand hygiene practice. Certain parts of the equipment, including pipes supplying water for cleaning, internal discharge tubes connected to discharge outlets and cup dispensers, are difficult to disassemble, clean, and sterilize, enabling microbial growth. On the other hand, microbial contamination of equipment parts that are easy to clean and sterilize was relatively low. Therefore, this should be taken into consideration by franchise headquarters when supplying food equipment to their franchisee stores (supply management, kind of management categories for securing systemic food safety; British Retail Consortium, 2011; Chilled Food Association, 2006). Moreover, many workplaces do not perform delicate tasks such as cleaning and sterilizing of discharge parts, and thus, guidelines may need to be refined. It is necessary to set a desorption cycle for single‐use gloves of workers in order to prevent microbial growth on hands due to sweat discharge. Alternatively, using single‐use gloves that repel organic matter, such as microbes and secretions, while also letting moisture out smoothly, should be considered.

Microbial survey studies of final food products based on evaluating compliance with legal standards are being widely conducted. However, it is difficult to determine underlying causes from these surveys, which makes establishing realistic countermeasures challenging.

The franchise industry is highly diversified (Park et al., 2019), and in many cases, small brands or franchisees, which are a type of franchise operation, often lack expertise in microbial management. Therefore, we simultaneously conducted microbiological analyses and third‐party audits to reveal fundamental and practical measures to be adopted and recommended solutions. It may be necessary to expand such research in the future.

## 4 CONCLUSION

In various countries, including Korea, the service‐based food industry—a kind of tertiary industry—is quickly developing and breaking away from the traditional manufacturing‐oriented secondary industries. One of the representatives of the service‐based food industry is the franchise food industry, which can be quickly developed through their collective operational strategies. Based on their collective operational natures, franchise food safety can be efficiently secured if a food safety management strategy optimized for the unique franchise operating environment is developed. Moreover, as the food franchise industry is newly developing, it must prepare optimized management standards for the present day. Therefore, this study analyzed their manufacturing processes of ten representative soft ice‐cream brads that are well representative of the characteristics of a franchise. Despite guidelines for food risk management being fundamentally organized and operated by every brand, microbial contamination or growth throughout the food processing procedure were confirmed. In particular, microbial load exceeded the Korean Food Safety Standard in the final product or working environment; this is due to insufficient cleaning and sterilization of handling equipment or packaging materials that are uniquely used in food franchise establishments and not in the traditional manufacturing business, which needs to be supplemented. In addition, although equipment suitable for the food franchise operation is used, the components of several device parts have an unfavorable structure for cleaning and disinfection, which requires systematic supply management during the equipment purchasing stage by franchise brand headquarters. Furthermore, as the cleaning and sterilization cycle of these devices or packaging materials in storing area are not yet set, it is necessary for them to supplement their own guidelines. Our findings suggest that in order to ensure collective microbial quality, cleaning, and sterilization regulations and practices should be updated and standardized.

## CONFLICT OF INTEREST

The authors declare that they do not have any conflict of interest.

## ETHICAL APPROVAL

This study does not involve any human or animal testing.
